# Advantages of low‐glycaemic‐index carbohydrates in regulating glycaemic response of energy bar: A review

**DOI:** 10.1002/jsfa.70775

**Published:** 2026-06-07

**Authors:** Raju Sasikumar, Ayush Gupta, Selva Kumar T, Kaviarasu Ganesan, Sheena Haorongbam, Ravikumar Rajarathinam, Sandeep Janghu, Sandeep Kumar Panda, Amit K. Jaiswal

**Affiliations:** ^1^ Department of Agribusiness Management and Food Technology North‐Eastern Hill University Tura, West Garo Hills India; ^2^ Vel Tech Rangarajan Dr. Sagunthala R&D Institute of Science and Technology Chennai India; ^3^ Center for Bioenergy and Bioproducts, Vel Tech Rangarajan Dr. Sagunthala R&D Institute of Science and Technology Chennai India; ^4^ Department of Food Technology Rajiv Gandhi University, Rono Hills Doimukh India; ^5^ School of Biotechnology, Campus 11, Kalinga Institute of Industrial Technology (KIIT) Deemed to be University Bhubaneshwar India; ^6^ School of Food Science and Environmental Health, Faculty of Sciences and Health, Technological University Dublin, City Campus Dublin Ireland; ^7^ Centre for Sustainable Packaging and Bioproducts, Technological University Dublin, City Campus Dublin Ireland

**Keywords:** energy bars, low‐glycaemic‐index carbohydrates, glycaemic response, inulin, fructooligosaccharides, sports nutrition, sustained energy release

## Abstract

Energy bars are widely consumed functional foods among athletes and physically active consumers who require convenient sources of sustained energy. Their carbohydrate composition, particularly the type and ratio of digestible sugars such as glucose, fructose and sucrose, strongly influences glycaemic index (GI), postprandial blood glucose response and subsequent metabolic outcomes. GI reflects the rise in blood glucose after food consumption relative to a reference carbohydrate – usually glucose. Low‐GI carbohydrates are digested and absorbed more slowly, resulting in a moderated insulin response and potentially greater reliance on fat oxidation during exercise. This review examines the effects of replacing high‐GI sugars with low‐GI and functional carbohydrate alternatives, including inulin, fructooligosaccharides (FOS) and sugar alcohols, in energy bar formulations. Evidence from formulation studies indicates that partial substitution of sucrose with inulin and FOS can reduce GI while maintaining energy density. For example, replacement of approximately 15% sucrose with inulin/FOS in papaya‐based fruit bars reduced GI from about 65 to 54, while energy density remained within 3260–3350 kcal kg^−1^. Similarly, granola bars formulated with coconut sugar, FOS or inulin instead of sucrose showed GI values in the low‐50 range. Such formulations may produce lower postprandial glucose and insulin responses, support steadier blood glucose availability and promote substrate utilization patterns favourable for endurance performance. The available evidence suggests that incorporating low‐GI carbohydrate blends into energy bars can improve their metabolic profile without substantially compromising caloric density. Standardizing sugar replacement strategies using ingredients such as inulin, FOS and selected sugar alcohols may support the development of energy bars with improved glycaemic control, sustained energy release and potential benefits for sports nutrition and metabolic health. © 2026 The Author(s). *Journal of the Science of Food and Agriculture* published by John Wiley & Sons Ltd on behalf of Society of Chemical Industry.

## INTRODUCTION

Currently, the rising awareness of ‘functional foods’ and ‘performance diet’ has contributed to the mounting popularity of energy bars, predominantly among athletes, fitness lovers and those looking for convenient sources of stable energy.^1^ Specifically, high‐energy vegan bars (HEVB) have become a preferred choice due to their health advantages, plant‐based components, and suitability for a broad audience.^2^ The fundamental principle of these energy bar formulations is the importance of carbohydrates, particularly sugars, which are vital macronutrients involved in energy metabolism, glucose regulation and overall energy yield.^3^

Carbohydrates (CHO) are the primary energy source in the human body. The process of carbohydrate metabolism, particularly sugars like glucose, fructose and sucrose, significantly impacts blood glucose management, insulin response and consequent energy availability.^4^ Structural distinction between monosaccharides, disaccharides and polysaccharides affects their metabolic behaviour and glycaemic response.^5^ After consumption, sugars undergo specific processes, including enzymatic breakdown, intestinal absorption and metabolism, predominantly through glycolysis and the tricarboxylic acid (TCA) cycle.^6^ Ultimately, those produce adenosine triphosphate (ATP), which acts as the body's usable energy currency.^7^

Premature exhaustion during an extended workout is associated with depletion of carbohydrate stores (muscle glycogen, blood glucose and liver glycogen). Hence, carbohydrate ingestion before, during and after the workout session enhances performance compared to an abstained state.^8^ Endurance or highly active categories of sports can be heavily affected by a depletion of glycogen storage. Soccer players often experience fatigue in the last 15 min of the game due to low glycogen levels, leading to reduced distance covered, sluggish sprints, and reduced capacity for acceleration and deceleration.^9^

Glycaemic index (GI) is a parameter that can differentiate carbohydrates and sugars on the basis of how quickly they can elevate glucose concentration in blood.^10^ Sugars with low GI cause a lower insulin response than sugars with high GI. Insulin hinders fat oxidation throughout exercise.^11^ Hence, lower GI sugars, if used in energy bars, can provide increased utilisation of fats over carbohydrates, which will facilitate the preservation of glycogen storage.^12^, ^13^, ^14^ Carbohydrates with low GI are known to support sustained energy release and enhance metabolic performance during prolonged exercise. In contrast, high‐GI carbohydrates are linked to rapid increases in blood glucose and insulin levels.^15^ Athletes often consume energy bars for stamina during exercise. The survey indicated that about 37% of soccer players consume functional foods, such as energy bars, an hour before the game to improve performance.^16^

The process of sugar absorption in the small intestine and the metabolic pathways involved reveal significant differences in the energy yield of various sugars. Transport mechanisms, such as sodium‐dependent glucose transporters (SGLTs) and facilitated diffusion, significantly affect the rate and efficacy of absorption, thereby influencing postprandial blood glucose concentrations and energy availability.^17^ Previous studies have revealed evidence of metabolic benefits (lower insulin levels, higher fat oxidation during exercise and lowered glycogen store depletion) when low‐GI sugar (fructose, inulin, etc.) is consumed before exercise or during intermittent activity. During these high‐intensity, exhaustive exercises (soccer, badminton, rugby, etc.), high‐fat oxidation can provide the energy needed by the muscles.^18^, ^19^

However, the efficiency of a high‐energy vegan bar intended for endurance athletes has barely been explicitly assessed. Therefore, this review explores the effects of varying sugar ratios on the glycaemic index and energy‐releasing potential of energy bars, examining both conventional sugars and new alternatives such as inulin, fructooligosaccharides (FOS), d‐tagatose and sorbitol. It also explains how sugars interact with other macronutrients, such as proteins and fats, in the management of energy expenditure and substrate oxidation. In addition, the review addresses the existing gaps in long‐term metabolic data, which must be filled by refining energy bar formulations to support sustained energy, metabolic efficiency and overall health benefits.

## CARBOHYDRATES AND ENERGY BALANCE

### Structure

Carbohydrates ${\left(CH_{2}O\right)}_{n}$ – the key energy‐producing biomolecules – are abundant in both plants and animals. The primary types of dietary carbohydrates are monosaccharides and disaccharides, such as fructose, glucose, lactose and sucrose, with starch being a polysaccharide.^20^, ^21^ The fundamental component of carbohydrates is monosaccharides. Two monosaccharides paired with a glycosidic bond are called disaccharides (sucrose, lactose), which have the general biochemical formula $C_{12}H_{22}O_{11}$. Oligosaccharides are polymers containing multiple monosaccharides – usually more than 2–10 (maltodextrins, raffinose). Polysaccharides are polymers composed of long, intricate chains of monosaccharides linked by glycosidic bonds (e.g., cellulose, amylose).^22^

### Classification of carbohydrates

Carbohydrates are mainly of four categories. Two or fewer monosaccharides and disaccharides linked in a straightforward structural arrangement form a simple carbohydrate. Simple carbohydrates can be rapidly used for energy, leading to a swift increase in blood sugar levels and triggering insulin release from the pancreas. Some simple carbohydrates include glucose, fructose, galactose, lactose, maltose, and sucrose.^23^ They are often found in foods like carbonated beverages, candies, corn syrup, and so forth. Complex carbohydrates, such as oligosaccharides and polysaccharides, are composed of more than two sugar molecules, arranged in more intricate structures. Their comparatively slower breakdown process causes a more gradual increase in blood sugar concentration. Cellobiose, amylose, cellulose, dextrin, etc. are complex carbohydrates. They are abundantly found in apples, brown rice, unrefined cereal grains, and so forth.^22^

Complex carbohydrates consisting of a large unit of glucose molecules are called starch, which is commonly present in plants and plant‐based products. It can be found in potatoes, chickpeas, pasta, and wheat.^24^ Being non‐digestible, fibres promote the beneficial bacteria's growth residing in the colon and work like a bulking agent in the facilitation of bowel movements. The primary elements are pectin, cellulose and hemicellulose. By remaining in the intestine, insoluble fibre softens the stool and facilitates regularity in bowel movements. Brans, seeds, vegetables and so forth are rich in insoluble fibre. On the other hand, soluble fibre helps to maintain blood cholesterol and low‐density lipoprotein levels. It also reduces the postprandial glucose spike. Fresh fruits, oats and beans are excellent sources of soluble fibre.^25^

### Metabolism

After ingestion, salivary amylase in the mouth initiates the breakdown of carbohydrates. As carbohydrates continue to be digested throughout the digestive tract, monosaccharides enter the bloodstream. After metabolism of a portion of ingested carbohydrates, which yield about 4 kcal g^−1^, the remaining carbohydrates are converted into glycogen, which is stored in the liver or muscles. About 400–500 g of carbohydrate can be stored as glycogen when the body is well fed.^26^ High blood sugar concentration caused by the ingestion of carbohydrates stimulates insulin secretion by the pancreas. Cells absorb glucose to yield energy or to store it under the influence of insulin. However, a reduction in blood sugar concentration stimulates glucagon secretion from the pancreas, leading to the release of stored glucose from the liver. The body cannot digest fibre, meaning it does not supply calories or energy. However, it offers various health benefits, such as adding bulk to stool for easier elimination, preventing constipation, possessing prebiotic properties, promoting a sense of fullness and addressing intestinal issues.^20^

Gut microbiota plays a significant role in regulating carbohydrate metabolism and insulin sensitivity, which influence overall energy balance. It contributes to the fermentation of indigestible carbohydrates, especially low‐GI fibre, producing short‐chain fatty acids (SCFAs) such as acetate, propionate and butyrate, which act as key metabolic regulators.^27^, ^28^ These SCFAs improve glucose homeostasis by enhancing insulin sensitivity in peripheral tissues and reducing hepatic glucose production.^27^ SCFAs stimulate the secretion of gut hormones such as glucagon‐like peptide‐1 (GLP‐1) and peptide YY (PYY), which regulate appetite and postprandial glycaemic response, linking carbohydrate quality to metabolic control.^29^, ^30^ Diets rich in low‐GI carbohydrates and dietary fibre promote beneficial microbial populations, especially butyrate‐producing bacteria, thereby improving metabolic efficiency and energy utilization.^28^

Dysbiosis is strongly associated with impaired insulin signalling and metabolic disorders. Studies show that individuals with insulin resistance or type 2 diabetes often exhibit reduced levels of beneficial bacteria (e.g., *Faecalibacterium prausnitzii*) and increased levels of opportunistic pathogens, leading to increased intestinal permeability and systemic inflammation.^27^ This condition promotes the translocation of lipopolysaccharides (LPS) into circulation, activating inflammatory pathways such as Toll‐like receptor signalling, which interferes with insulin receptor activity and downstream signalling molecules such as IRS and Akt.^28^ Additionally, dysbiotic microbiota can enhance energy harvest from carbohydrates, increasing fat deposition and contributing to insulin resistance.^28^, ^31^ Consequently, modulation of gut microbiota through low‐GI, fibre‐rich vegan formulations may serve as an effective nutritional strategy to improve insulin sensitivity and maintain metabolic homeostasis.

## CARBOHYDRATE METABOLISM AND METABOLIC FLEXIBILITY IN SKELETAL MUSCLE

Skeletal muscle at rest uses a mixture of fats and carbohydrates for energy, whereas during intense exercise it relies on muscle glycogen and blood glucose. Metabolic flexibility involves the ability to switch between fat oxidation at low intensity and carbohydrate oxidation at high intensity, which is crucial for athletes (and by extension consumers of energy bars) because it maximizes endurance and recovery.^32^

### CHO uptake of muscle at rest *versus* exercise

At rest, muscle glycogen phosphorylase is mostly inactive and insulin‐mediated glucose uptake is moderate. In this state energy comes from both carbohydrate and fat oxidation (blood glucose, plasma free fatty acids and stored glycogen/triacylglycerols are major fuels).^32^, ^33^ When exercise begins, muscle contraction triggers Ca^2+^ release and increases the AMP/ADP ratio, activating AMPK and phosphorylase kinase; this rapidly converts phosphorylase to its active form and accelerates glycogenolysis.^34^ As a result, glucose uptake into muscle spikes via insulin‐independent GLUT4 translocation (exercise directly increases GLUT4 activity), which explains how exercise shifts muscle to burn glycogen (via glycolysis) to produce ATP, whereas at rest glycogen breakdown is low.^32^, ^34^

### Metabolic flexibility and practical relevance

Study showed that training and workload strongly influence substrate use. In effect, a trained muscle can increase fatty acid use at submaximal effort and rely on carbohydrate when intensity rises, illustrating metabolic flexibility. It was noted that exercise intensity, duration and prior carbohydrate availability are major modulators of muscle glycogen use. This flexibility matters for athletes and for formulators of high‐energy bars. It means that the body can adapt to available fuel. After exercise, muscle insulin sensitivity is increases, so any ingested carbohydrate (especially high‐GI sugars) is quickly taken up for glycogen resynthesis.^35^

### Sugar types and bar formulation implications

High‐glycaemic (rapid) sugars (e.g., glucose, sucrose) are digested quickly, causing a quick spike in blood glucose and insulin that support rapid muscle glucose uptake. By contrast, low‐GI (slow) carbohydrates release glucose more gradually,^36^ whereas, through a study, it was reported that during exercise ingested carbohydrate can be oxidized at ~1.8 g min^−1^ (with added sucrose), suggesting that including quickly oxidizable sugars in a bar can directly fuel working muscle.^37^ In practice, bars consumed during or immediately after exercise should emphasize fast carbohydrates to match muscle's elevated uptake (since insulin‐independent glucose uptake is high), while slower‐digesting ingredients (fibre, complex carbohydrates) can be included for sustained energy.^34^

## MECHANISM OF ABSORPTION AND METABOLISM OF SUGAR

### Absorption

The primary sugars consumed by an individual include glucose, fructose and sucrose. The amount of lactose varies, influenced by dairy product intake. However, lactose is increasingly being incorporated into certain products that are not dairy based.^38^ Maltose, a disaccharide, occurs in small amounts in products based on cereals but can be found in greater quantities in isomaltose, alongside glucose syrups and the larger oligosaccharides from the maltose series. Monosaccharide sugars, being small in size, can diffuse through mucosal cell membranes (Fig. 1); thus, one possible way they can be absorbed is via a concentration difference, leading to indirect diffusion. Glucose is not the primary sugar entering the small intestine. However, disaccharides (maltose, sucrose and lactose) are broken down and converted into glucose, making glucose the predominant carbohydrate in the enterocyte, which is then transported into the body.^39^

**Figure 1:**
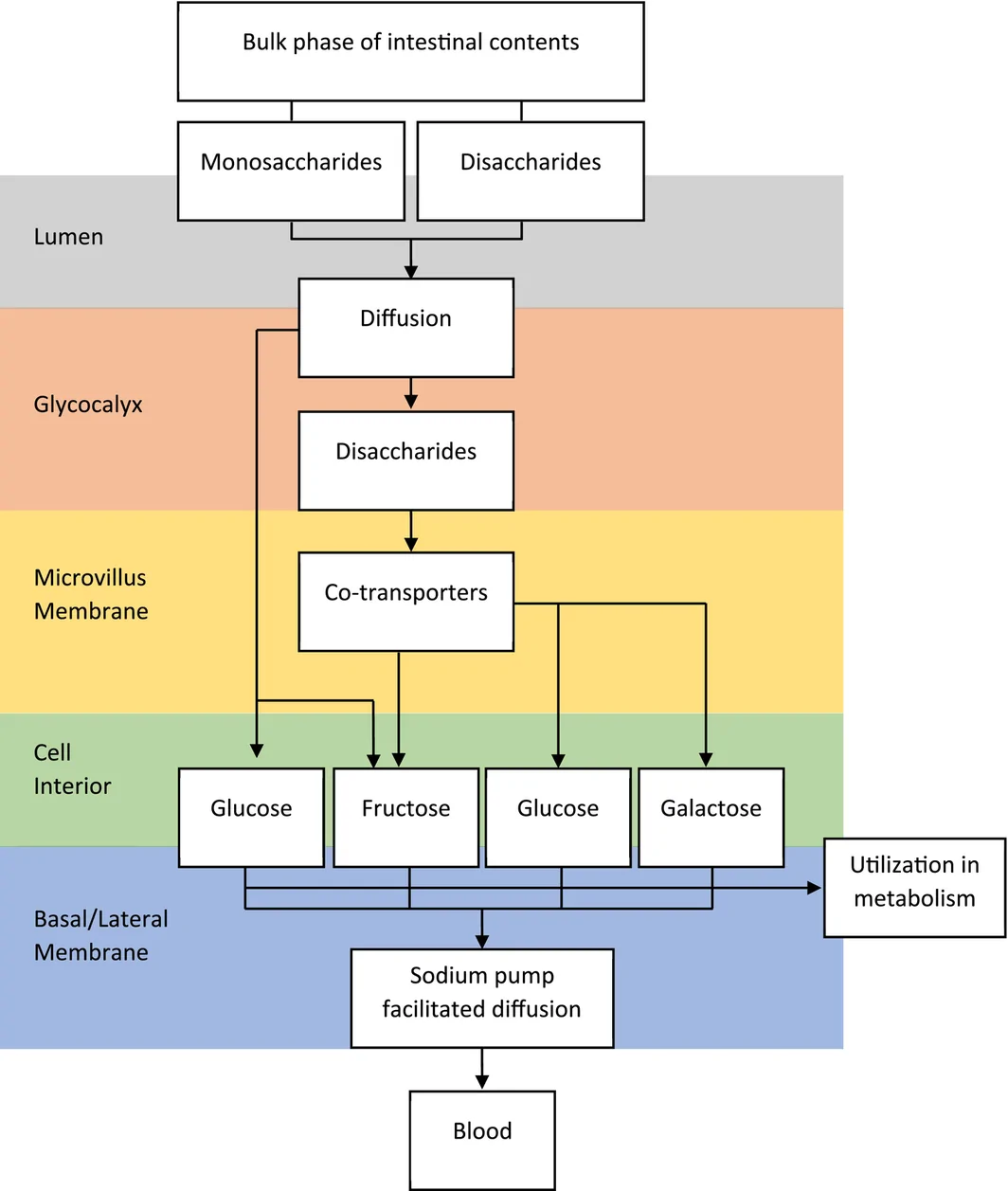
Phases of sugar absorption from small intestine to blood.

A sodium‐mediated co‐transporter system facilitates glucose absorption.^17^ In different locations of the small intestine, varying amounts of co‐transporter sites are present, the duodenum and upper jejunum being most prevalent and functioning at their highest capacity. Research indicates that providing carbohydrate‐rich diets in mice can stimulate the co‐transporter system, and eliminating carbohydrates from their diet will make the system less active.^40^ A structural specificity – the structure of pyranose where the first carbon should have a hydroxyl group and the sixth carbon should have a methyl group or a derivative of methyl – is required by the transporter system for carbohydrates. Although the binding sites for sodium and glucose are spatially distinct, the binding of sodium ions induces conformational changes that enhance the glucose binding affinity. A minimum of four distinct transporter systems is required to account for glucose and galactose transport, as determined by a kinetic analysis of vesicle preparations.^41^ Although some species display indications of fructose being actively transferred by an energy and sodium‐dependent pathway that is different from the glucose–galactose co‐transporter, it is believed that fructose is absorbed through a carrier‐mediated diffusion. The absorption of fructose when consumed with sucrose or alongside glucose seems to be more efficient than when fructose is ingested on its own.^42^

Due to their large molecular structure, disaccharides cannot cross the mucosal cell membrane unless they are hydrolysed before absorption (Fig. 1). In the small intestine, the transportation of glucose/galactose and fructose occurs from the lumen to the enterocyte to the blood, where enterocytes have two sides: the brush border membrane, which faces the lumen, and the basolateral membrane, which faces the blood. Glucose and galactose are transported via sodium–glucose linked transporter 1 (SGLT1); this is done by secondary active transport, where Na^+^ and glucose/galactose enter the cell together due to a gradient.^43^ In transportation, Na^+^/ K^+^‐ATPase pumps play a significant role as an energy provider, which are located on the basolateral membrane. They pump Na^+^ out and K^+^ in, which maintains a low sodium concentration inside the cell, allowing sodium to drive SGLT1 to keep working.^44^ Then comes GLUT5, which facilitates fructose diffusion along the concentration of gradient without energy or passively. Lastly, GLUT2, which is also located on the basolateral membrane, transports glucose, galactose and fructose out of the cell into the blood through diffusion, as shown in Fig. 2.^45^, ^46^ Sucrose, a β‐d‐fructofuranosyl‐α‐d‐glucopyranoside, is a primary disaccharide found in most human diets. In a Western diet, individuals may consume up to 100 g per day.^39^ Research shows that disaccharides are broken down and their resulting products (such as glucose) are absorbed at rates greater than that of direct glucose transport. The only mechanism restricting the speed at which monosaccharides of lactose are absorbed is the hydrolysis of lactose.^47^

**Figure 2:**
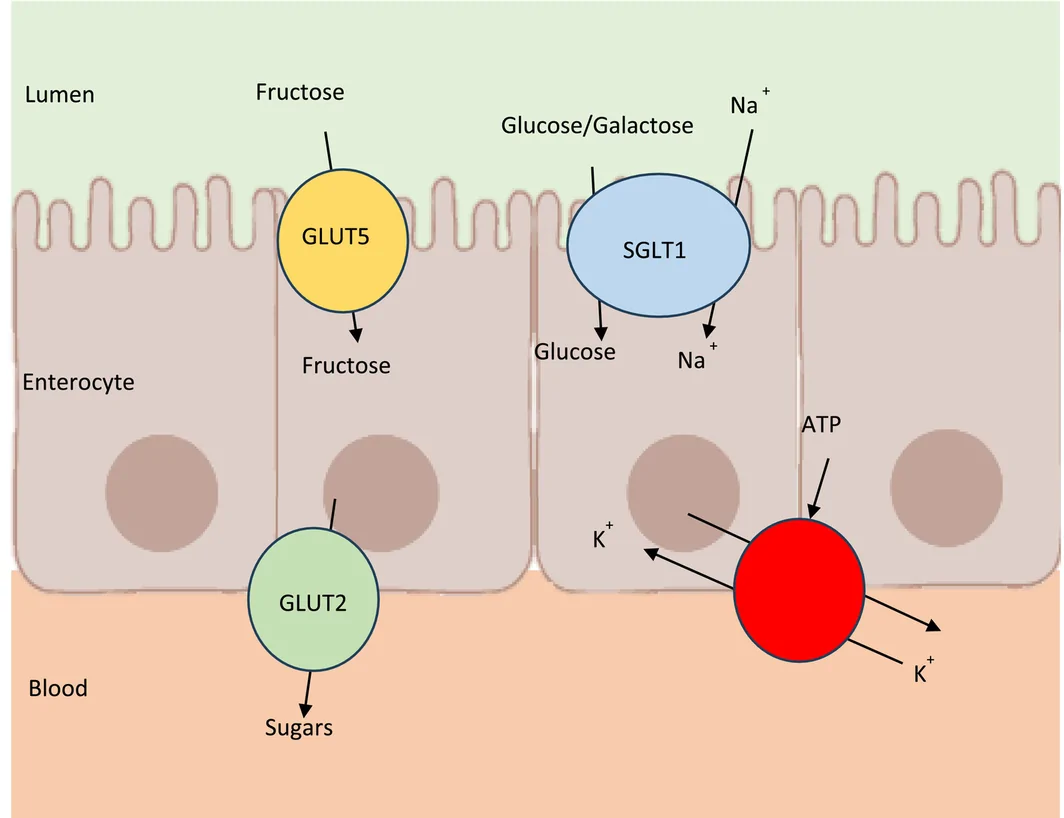
Classical model of sugar transport in the intestine illustrates that SGLT1 is a sodium‐dependent transporter for glucose and galactose situated on the brush border membrane (BBM). The Na^+^K^+^‐ATPase located on the basolateral membrane (BLM) is responsible for maintaining the gradient essential for the effective operation of SGLT1. On the BBM, GLUT5 serves as a transporter that facilitates the entry of fructose into the cell. GLUT2, found on the BLM, is responsible for moving glucose, galactose, and fructose from the cell into the bloodstream.

### Metabolism

From all the monosaccharides existing in carbohydrates present in the diet, primarily three enter the bloodstream after digestion and absorption. Glucose is obtained directly or, for most diets, after hydrolysis of disaccharides (lactose, sucrose, etc.) and starch. Therefore, approximately 214, 50, and 8 g of glucose, fructose and galactose, respectively, are included in a typical adult's regular diet, and the carbohydrates consumed converge in their metabolic pathway at the transport systems located in the mucosal membrane.^48^ The metabolic processes utilized by the three monosaccharides share certain standard features. Ultimately, carbon dioxide and water are produced from the monosaccharides through the TCA cycle.^49^

Two pyruvate molecules are produced from one glucose molecule during glycolysis – the initial phase of glucose degradation, which is a six‐step metabolic process. The pyruvate molecules subsequently enter the TCA cycle (Fig. 3). During glycolysis, two ATP molecules are exhausted to generate four ATP molecules. Hence, this process gains two ATP molecules.^6^ Ultimately, a total of 36 ATP molecules is generated by the complete oxidation of a glucose molecule through both the glycolytic pathway and the TCA cycle.^50^

**Figure 3:**
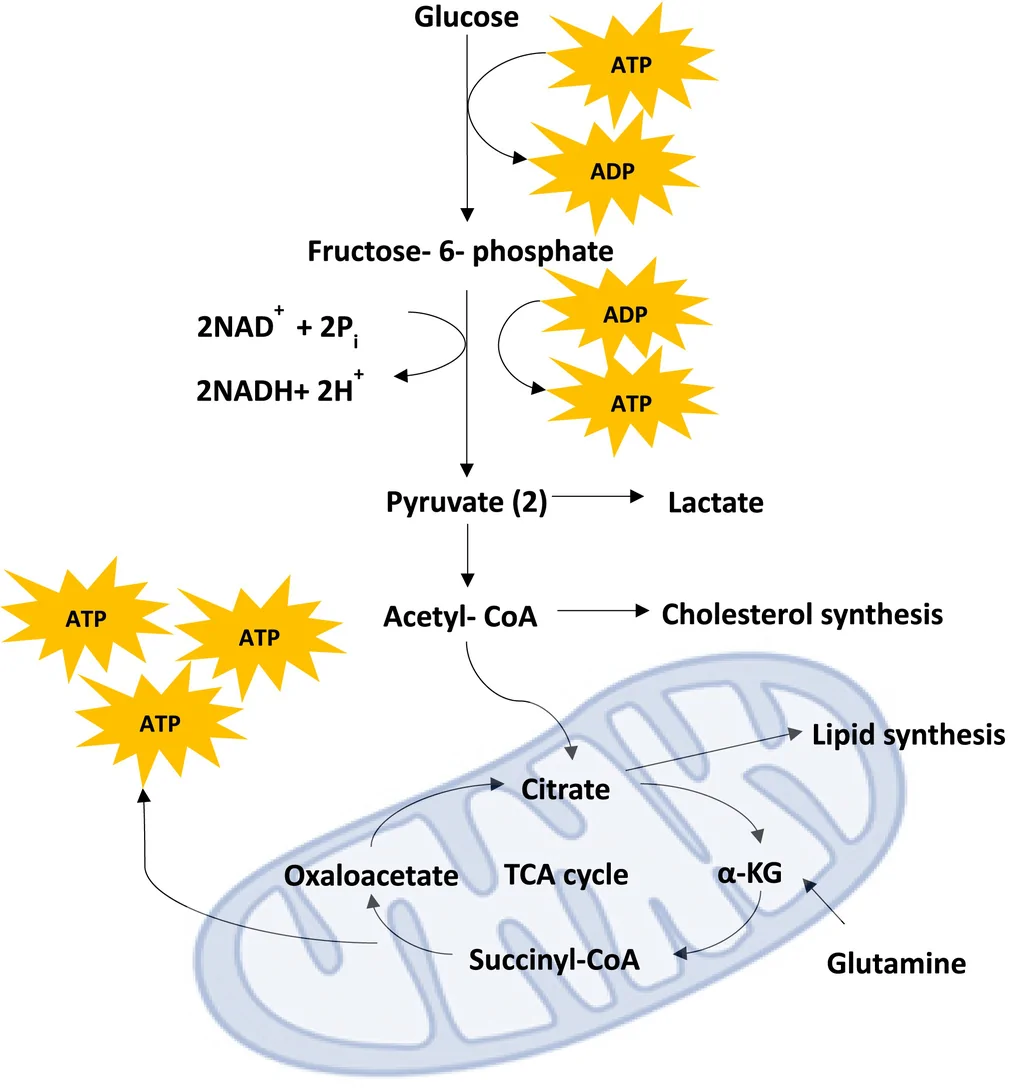
Metabolism of sugar.

The metabolism of fructose forms fructose‐1‐phosphate, which gradually becomes divided into dihydroxyacetone phosphate and glyceraldehyde. The former is required in gluconeogenesis and glycolysis. The latter is then phosphorylated and combined again with the former to form fructose‐1,6‐bisphosphate, which eventually contributes to glycogen synthesis.^51^ The primary metabolic consequences of fructose consumption affect the glycolytic pathway, resulting in increased production of pyruvate and lactate. This process reduces fatty acid oxidation while promoting the synthesis and esterification of very‐low‐density lipoprotein.^52^ However, the enzymes galactose‐1‐phosphate uridyl transferase and uridyl diphosphate epimerase convert galactose into galactose‐1‐phosphate, which is progressively converted into glucose‐1‐phosphate. This glucose‐1‐phosphate can either be stored as glycogen or undergo further metabolism.^53^, ^54^

## FUNCTIONS

Glycolysis, gluconeogenesis, glycogenolysis and glycogenesis are essential biological processes related to glucose metabolism. In the liver, glycolysis facilitates glucose metabolism at the cellular level through a series of enzymatic reactions. The liver can monitor serum glucose levels and utilize glucose when it is elevated after a meal, using an enzyme called glucokinase.^6^ During fasting periods, when there is no intake of glucose (e.g., overnight while sleeping), gluconeogenesis occurs.^55^

Gluconeogenesis is the process by which mitochondria of liver cells produce glucose from non‐carbohydrate sources.^56^ In addition, during times of fasting, the pancreas releases glucagon, which initiates glycogenolysis. In glycogenolysis, glycogen – the stored form of glucose – is broken down and released as glucose.^57^ Glucose tolerance is influenced by the circadian rhythm. Typically, people experience their highest glucose tolerance in the morning. In contrast, oral glucose tolerance tends to be lowest in the afternoon and evening. This low point is likely because pancreatic β cells are most responsive in the morning, while glycogen storage is maximized in the evening. Insulin sensitivity in adipose tissue is most significant in the afternoon.^58^

## SUGAR AND GLYCAEMIC INDEX

The changes in blood glucose response after consuming the same amount of carbohydrates from various dietary sources, which potentially affect an individual's well‐being, health and performance, constitute the basis of GI.^59^ After consumption of 50 g of accessible carbohydrates from test food, the GI is defined as the incremental area under the blood glucose curve (0–2 h), expressed as a percentage of the corresponding area after ingesting an equivalent amount of carbohydrate from a reference food. Commonly, a high GI is associated with a strong insulin response.^60^

Research has shown that consuming carbohydrates with low GI is better than consuming those with high GI prior to exercise, as they can enhance fat oxidation while reducing carbohydrate oxidation.^61^ Eight participants ingested 1.5 g kg^−1^ of accessible carbohydrates from a lentil‐based bar with low GI (45) or a bar with high GI (101), a couple of hours before a 90 min experimental soccer game, along with 0.38 g kg^−1^ body weight of the same during the 15 min half‐time interval. Throughout the game, carbohydrates were oxidized significantly more slowly (*P* < 0.05) with the low‐GI bar (2.17 ± 0.6 g min^−1^) than with the high‐GI bar (2.72 ± 0.4 g min^−1^).^15^ It was observed that a bar with low GI (lentil‐based) offers a metabolic advantage (carbohydrate oxidation ↓) and a slight enhancement in tests of agility and jumping height towards the end of the test.^15^ Similar results were observed in a study that examined the effect of ingesting a low‐GI lentil‐based sports energy bar prior to exercise, compared with a commercial sports energy bar with a moderate glycaemic index, on performance outcomes during endurance cycling.^18^

In a previous study, the GI of a bar branded ‘Nothing Else’ was evaluated using 24 fit participants. It was found to be lower than that of two popular New Zealand commercial snack bars. The ‘Nothing Else’ bar and commercially available bars 1 and 2 contained 81, 83, and 151 g kg^−1^ of sugar, respectively. Half an hour after ingestion, the increase in blood glucose concentration for the ‘Nothing Else’ bar (6.1 ± 0.7 mmol L^−1^) was lower compared to bar 1 (6.7 ± 0.9 mmol L^−1^) and bar 2 (7.1 ± 0.9 mmol L^−1^). The increase in blood glucose over 45 min following the consumption of the 'Nothing Else' bar appeared to occur more slowly and to a lesser extent than that of the other two bars.^19^

Earlier research assessed how various foods affect blood glucose levels. Sixty‐two frequently ingested sugars were independently administered to groups of five to ten healthy fasting participants. Concentrations of glucose in the blood were recorded over a 2 h period and expressed as a percentage of the area under the glucose response curve to an equivalent carbohydrate intake. The highest increases in blood glucose were observed with vegetables, and dried legumes were the lowest.^19^ An important negative correlation was observed between fat and protein and the rise in postprandial glucose, while no significant relationship was detected with fibre or sugar levels. Notable variations were found not only between the food groups but also within many of them; for instance, amongst cereals, the GI for wholemeal bread was 72%, while it was 42% for wholemeal spaghetti; and among root vegetables, parsnips scored 97% compared to sweet potatoes at 48%. A remarkable observation was that carbohydrate‐rich foods with the lowest GI (oatmeal porridge, spaghetti, buckwheat, yams, sweet potato) were frequently consumed by lower‐income populations in Western nations and in large areas of Africa and Asia.^62^

In a study, the highest average GI values were recorded for potatoes (58% high‐GI foods), rice (38% high‐GI foods), sweet potatoes (52% high‐GI foods), and Asian–Indian cuisine (37% high‐GI foods). In contrast, the lowest outcomes were observed in meal replacement products (100% low‐GI items), dairy products (95% low‐GI items), legumes (94% low‐GI items), and sports energy bars (94% low‐GI items). Nuts had the lowest average GI values (100% low‐GI items). Overall, savoury snacks had greater GI values (60 ± 15; 46% low GI items and 34% high GI items) compared to sweet snacks and confectionery products (48 ± 16; 68% low‐GI items and 11% high‐GI items).^63^

Regulation of carbohydrate intake plays a significant role in controlling satiety through both physiological and metabolic mechanisms. Carbohydrate‐rich foods, especially those with low GI, promote a more stable postprandial glucose response, thereby preventing rapid spikes and declines in blood glucose that are related to increased hunger.^64^ Low‐GI carbohydrates also enhance satiety by stimulating the release of gut‐derived peptides such as GLP‐1, PYY and cholecystokinin, which act on the brain to reduce appetite and prolong the feeling of fullness.^65^, ^66^ Additionally, the presence of carbohydrates in the gastrointestinal tract leads to gastric distension and nutrient sensing in the small intestine, both of which generate neural and hormonal signals that limit meal size and regulate energy intake.^65^ From this it can be stated that the incorporation of complex and fibre‐rich carbohydrates in vegan bars can enhance satiety by increasing bulk, reducing energy density, and slowing digestion, thereby improving appetite control.

Carbohydrate composition also influences gut transit time and digestive kinetics, which are important determinants of energy balance. Slowly digestible carbohydrates and dietary fibre delay gastric emptying and intestinal transit through mechanisms such as the ‘ileal brake’, whereby the presence of undigested nutrients in the distal intestine triggers feedback signals that slow gastrointestinal motility.^65^ Hormones like GLP‐1 and PYY, released in response to carbohydrate ingestion, further contribute to reduced gastric emptying and prolonged intestinal transit, allowing for sustained nutrient absorption and prolonged satiety.^66^ Conversely, rapidly digestible high‐GI carbohydrates accelerate glucose absorption and gastric emptying, leading to shorter satiety duration and faster return of hunger.^64^ Fermentable carbohydrates such as resistant starch and soluble fibre can regulate colonic transit and produce SCFAs, which eventually help to regulate peristalsis and appetite.^30^, ^67^ Optimizing carbohydrate quality in energy bars by incorporating low‐GI and fibre‐rich ingredients can effectively regulate satiety and gut transit, contributing to improved metabolic health.

## VARIOUS SUGAR RATIOS USED IN ENERGY BARS AND THEIR EFFECT ON RELEASING ENERGY

Sugar ratios used in the formulation of any recipe of food articles influence the energy release caused by consumption of the food article. Research shows that a high‐energy cereal bar (HCB) formulated from puffed rice, fruits and sweetener is of a higher total energy value (4813.5–6798.7 kcal kg^−1^) than cereal bars made from almonds and baru pulp (3373.7 kcal kg^−1^) or pseudo‐cereal‐incorporated gluten‐free cereal bars (1803.9 kcal/kg).^68^ The HCB recipe exhibiting the lowest gross energy (4813.5 kcal kg^−1^) had a cereal, fruit and sweetener ratio of 60:14:26. In comparison, another recipe that showed the highest gross energy (6798.7 kcal kg^−1^) had a cereal, fruits and sweetener ratio of 60:17:23. The HCB was prepared with puffed sungyod rice (199.5 g kg^−1^), puffed Mue Lor rice (199.5 g kg^−1^), Bambara nut (133 g kg^−1^), white sesame seed (66.5 g kg^−1^), and black sesame seed (66.5 g kg^−1^), fruits like Saba chips (67 g kg^−1^g) and dates (67 g kg^−1^g), and sweeteners like corn syrup (100.5 g kg^−1^g) and honey (100.5 g kg^−1^g). The HCB contained 101.3–391.9 g kg^−1^ of total sugars. It was observed that the addition of cereals along with fruits and sweeteners increased the overall sugar content.^68^

Papaya and banana bars were developed in a study that included FOS and inulin at different ratios, available in both liquid and powder forms. The control bars were formulated with a composition of 700 g kg^−1^ fruit pulp, 300 g kg^−1^ sucrose, and 3 g kg^−1^ citric acid, while the modified bars consisted of 700 g kg^−1^ fruit pulp, 150 g kg^−1^ sucrose, 150 g kg^−1^ inulin and FOS, and 3 g kg^−1^ citric acid. A mixture of 700 g kg^−1^ papaya fruit pulp with 300 g kg^−1^ sucrose and 700 g kg^−1^ banana pulp with 300 g kg^−1^ sugar was established as the standard, yielding 3302 and 3404 kcal kg^−1^ of energy, respectively. In contrast, the modified bar made with 700 g kg^−1^ papaya fruit pulp, 150 g kg^−1^ sucrose, 135 g kg^−1^ powdered FOS, and 15 g kg^−1^ inulin resulted in an energy content of 3266 kcal kg^−1^. Another variation using 700 g kg^−1^ banana fruit pulp, 150 g kg^−1^ sucrose, 135 g kg^−1^ liquid FOS, and 15 g kg^−1^ kg^−1^ generated 3354 kcal kg^−1^ of energy.^69^

Granola bars were made employing barley flakes, groundnut pieces, cocoa powder, and either sucrose or substitutes such as coconut sugar, FOS, and inulin. The formulation that excluded sucrose had a carbohydrate content of 225 g kg^−1^ and approximately 2670 kcal kg^−1^ of energy.^70^ Conversely, the mixture that included FOS contained 360 g kg^−1^ carbohydrates and provided 3510 kcal kg^−1^ of energy. For the formulation with inulin, the carbohydrate content was 267 g kg^−1^, yielding 3145 kcal kg^−1^ of energy. The mixture containing coconut sugar had 433 g kg^−1^ carbohydrates and delivered 3818.5 kcal kg^−1^ of energy. Lastly, the formulation that included sucrose contained 431.3 g kg^−1^ carbohydrates and provided 3787.4 kcal kg^−1^ of energy (Table 1).^70^ No notable differences were observed in the carbohydrate levels and caloric content of granola bars produced using coconut sugar compared to those made with sucrose. However, the carbohydrate content and caloric value were higher in granola bars containing coconut sugar, followed by sucrose, FOS, and inulin, and were lower in those without sucrose.^70^

**Table 1:** Proximate composition and calorific value of granola bars made with various sucrose alternatives and without sucrose^70^

Formulation	Proximate composition (g 100 g^−1^)						Calorific value (kcal)
	Moisture	Carbohydrates	Proteins	Fats	Crude fibre	Ash	
Without sucrose	32.37	22.51	14.38	16.71	12.62	1.41	266.90
FOS	9.15	36.05	14.49	16.54	22.42	1.36	351.01
Inulin	25.12	26.71	14.57	16.33	16.23	1.00	314.52
Coconut sugar	9.74	43.33	14.70	16.64	13.70	1.90	381.85
Sucrose	9.40	43.13	14.55	16.43	14.95	1,51	378.74

Abbreviation: FOS, fructooligosaccharides.

In a research study, inulin and d‐tagatose were utilized as alternatives to sucrose in recipes for dark chocolate. The ratios of inulin to d‐tagatose in the chocolate blends were 100:0, 75:25, 50:50, 25:75, and 0:100. The control, which consisted of 100 g sucrose, produced 5236 kcal kg^−1^ of energy. Conversely, the formulations containing 100 g inulin, 75 g inulin combined with 25 g d‐tagatose, 50 g inulin mixed with 50 g d‐tagatose, 25 g inulin and 75 g d‐tagatose, and 100 g d‐tagatose generated energy values of 3796, 3835, 3874, 3912, and 3951 kcal kg^−1^ respectively.^71^

Inulin is a type of plant polysaccharide that, because of its chemical composition, cannot be broken down by human digestive enzymes but can be processed by certain bacteria present in the human microbiome, functioning as a prebiotic. As a result, the intake of inulin has been linked to alterations in the gut microbiota, which are associated with improvements in metabolic health and help mitigate various obesity‐related issues.^72^ Inulin has the structure of a linear polysaccharide consisting of fructose units linked by β‐2,1 glycosidic linkage, typically with a glucose moiety connected at the end with an α‐1,2 glycosidic linkage.^73^ Inulin has been widely used in certain food products due to its functional properties (Table 2). In addition, the primary mechanisms of inulin are shown in Fig. 4.

**Table 2:** Applications of inulin in foods

Application	Function	Concentration level (%, w/w)	Reference
Bakery foods	Prebiotic and fibre Preservation of moisture Sugar replacement	2–15	87
Breakfast cereals	4 Fibre and prebiotic 5 Crispiness 6 Increase in size.	2–25	88
Dairy products	7 Replacement of fat and sugar 8 Texture 9 Mouthfeel 10 Foam stabilization 11 Fibre and prebiotics	2–10	89
Frozen desserts	12 Substitution of fat and sugar 13 Enhances consistency 14 Melting characteristics 15 Fibre and prebiotics 16 Reduced caloric value	2–10	90
Chocolate	17 Replacement of sugar and fat 18 Heat resistance (melting behaviour)	5–30	91

**Figure 4:**
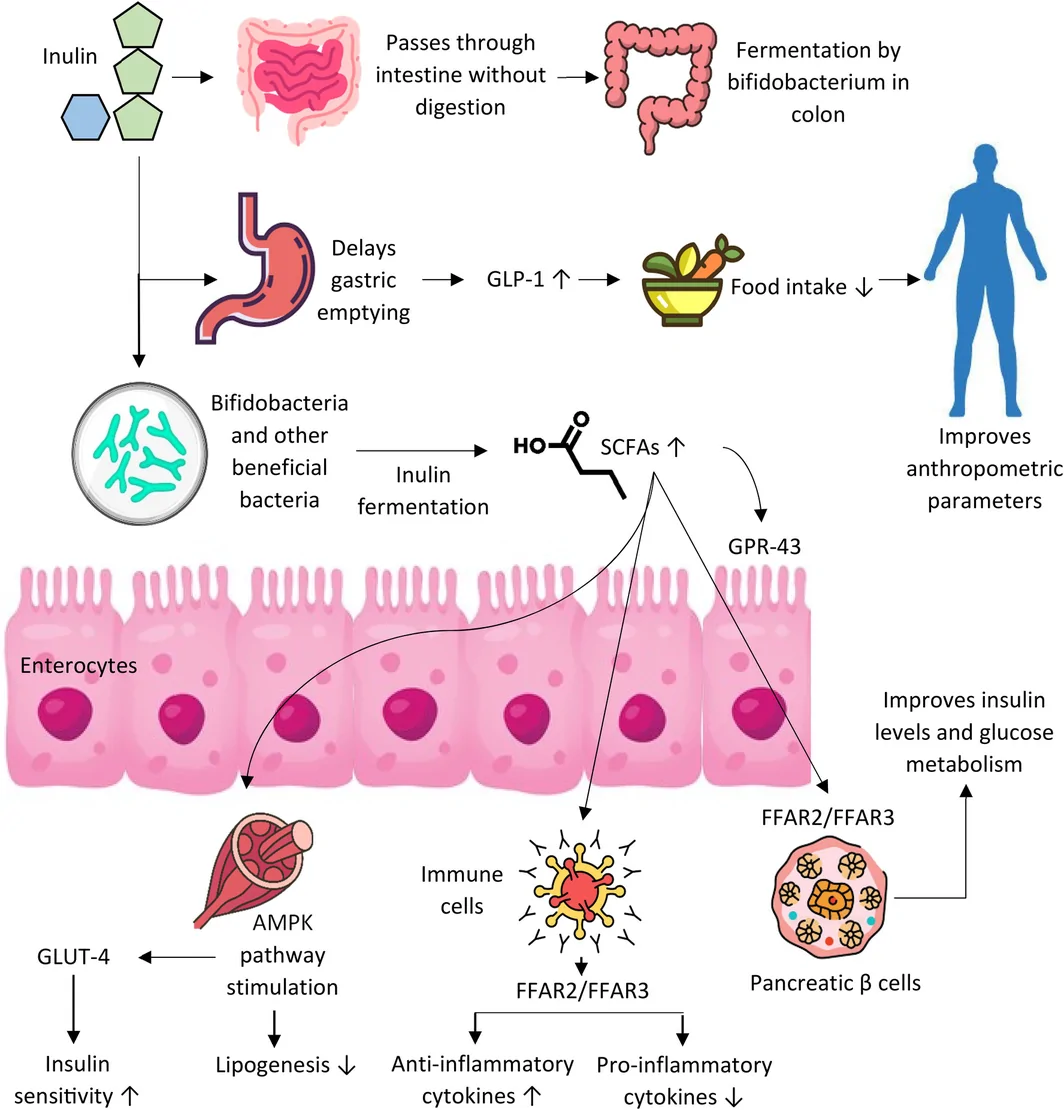
Overview of the primary mechanisms of action and physiological effects of inulin. Glucagon‐like peptide‐1 (GLP‐1), short‐chain fatty acids (SCFAs), glucose transporter 4 (GLUT‐4), G‐protein‐coupled receptor 43 (GPR‐43), free fatty acid receptors 2 and 3 (FFAR2, FFAR3), AMP‐activated protein kinase pathway (AMPK pathway).

Previous research employed RSM to investigate how different ratios of sorbitol, inulin and acacia gum affect energy production characteristics. Seventeen experiments were carried out with varying ratios of sorbitol, inulin and acacia gum. The fifth formulation, which included 48.7 g sorbitol, 20 g acacia gum and 5.7 g inulin, produced an energy yield of 3389 kcal kg^−1^. Similarly, the 11th formulation, comprising 38 g sorbitol, 16.6 g acacia gum and 14 g inulin, also achieved an energy yield of 3389 kcal kg^−1^. No significant differences in caloric values were observed among the formulations. The total sugar content of the fifth formulation was statistically different from the 11th formulation, ranging from 505.8 to 600.2 g kg^−1^. The reducing sugar content was statistically different (*P* ≤ 0.05), with values ranging from 51.7 g kg^−1^ in the 11th formulation to 62.2 g kg^−1^ in the fifth formulation.^74^ Overall, the impact of sugar ratios on energy release in energy bars is examined in the aforementioned articles, which used different types of sugar in various formulations. However, mixed results were obtained related to a linear relationship.

## RELATION BETWEEN RATIOS OF OTHER MACRONUTRIENTS WITH SUGAR

Research on diet‐induced thermogenesis indicates that when fats are digested, fewer calories are released (≈7%) than when proteins (≈22%) or carbohydrates (≈12%) are digested.^75^ An investigation into the impact of various carbohydrates on post‐meal thermogenesis over a 6 h period revealed that consumption of glucose (8%) and starch (8.2%) gave a lower thermogenic response compared to fructose (11.1%) and sucrose (11.4%).^76^ In another similar study, the data were comparatively lower, ranging from 10% (glucose) to 17% (sucrose or fructose and glucose).^77^ Changes in the levels of dietary macronutrients will influence the types of metabolic fuels utilized, though the impact on 24 h energy expenditure (EE) remains minimal until the dietary exchange is isoenergetic.^78^ Alteration of the carbohydrate proportion of food intake from 9% to 79% with equivalent reductions in fat consumption did not affect the 24 h EE assessed through whole body calorimetry.^79^ Isoenergetic exchange had no significant impact on 24 h EE whether the surplus was given as carbohydrate (approximately 215 kcal per day) or fat (≈96 kcal per day) in a situation of 50% overfeeding.^80^

Macronutrients are metabolized in an oxidative order, with alcohol taking precedence and fat given the least priority. This is significant, as growing evidence suggests that achieving energy balance is closely tied to maintaining a balanced intake of macronutrients.^81^ The regulation of protein intake over a broad spectrum is well established in humans. Therefore, maintaining body weight is not mainly reliant on protein balance. To sustain carbohydrate balance, it is essential to determine whether the conversion of glucose into fatty acids is an active process. If a substantial amount of carbohydrate can be transformed into fat, the idea of energy balance relying on carbohydrate and fat balance would lose its relevance.^82^

Since the human body can only store a limited amount of carbohydrates (approximately 300–500 g glycogen), any surplus should essentially be either oxidized or transformed into fat through de novo hepatic lipogenesis.^83^ In a recent study investigating the impact of meals rich in sucrose *versus* meals rich in starch on fuel selection, it was discovered that, after consuming a meal rich in sucrose, fructose is preferentially utilized compared to glucose. Additionally, it was found that glucose is metabolized at a slower rate following a sucrose‐rich meal than following a starch‐rich meal.^84^ It was previously observed that a group of individuals who switched their diet from low in fats and high in carbohydrates to high in fats and low in carbohydrates, paired with or without exercise for glycogen depletion, were able to quickly (within 24 h) adapt their fat intake to fat oxidation.^85^ It was noted that, as total energy expenditure rises, similar to what is seen in athletes, additional energy consumption is taken primarily from carbohydrates, with a specific inclination towards sugars.^86^

## CONCLUSIONS

Despite extensive research on the relationships among sugar ratios, glycaemic index, and energy release in experimental settings, unresolved questions remain about how sugar ratios compare with other dietary components in energy bars with respect to total energy release in individuals. To understand the potential confounding effects of other food components on energy release, as well as the types of food consumed (liquid or solid), more detailed research is needed. There are limited data on physiological responses over 12–60 h following the consumption of a product by a group of individuals, specifically regarding thermogenesis and levels of satiety. Estimating the long‐term effects on thermogenesis and energy expenditure requires an understanding of compensatory effects over 24 or 48 h. In addition to sucrose, various sugars are commercially employed in the production of energy bars. However, the effects of these sugars and their ratios in energy bars, along with physiological data on blood glucose levels, insulin response, weight gain and thermogenic effects, have not yet been thoroughly examined in existing studies. The relationship with diet, particularly the different carbohydrate types and the ratio of carbohydrates to fat, is barely researched. Another area of interest is how carbohydrates function in this interaction related to body weight management. The sugar composition in energy bars is crucial for influencing glycaemic response and energy release. Sugars with a low GI (fructose) are known to promote fat oxidation and help maintain energy levels during exercise. In contrast, high‐GI sugars (glucose) lead to a rapid energy increase followed by a decline. Differences in sugar proportions across formulations significantly affect calorie yield and metabolic outcomes. Incorporating functional sugars such as inulin, FOS, and d‐tagatose into energy bars offers options for reducing the glycaemic effect while maintaining energy density. Yet, the potential for energy release is determined not only by the type of sugar but also by its interactions with other macronutrients and the overall food structure. Sources high in complex sugars and fibre may offer improved satiety and a more gradual release of energy. In summary, standardisation of sugar ratios can help align energy bars with health and performance objectives.

## AUTHOR CONTRIBUTIONS

Raju Sasikumar: conceptualisation; validation; supervision; methodology; investigation; project administration. Ayush Gupta: writing – original draft; formal analysis; data curation. Selva Kumar T: writing – review and editing, writing – original draft. Kaviarasu G: writing – review and editing. Sheena Haorongbam: writing – review and editing. Ravikumar Rajarathinam: writing – review and editing. Sandeep Janghu: writing – review and editing. Sandeep Kumar Panda: writing – review and editing. Amit K. Jaiswal: conceptualisation, validation, writing – review and editing.

## CONFLICT OF INTEREST

The authors declare that they have no known competing financial interests or personal relationships that could have influenced the work reported in this paper.

## FUNDING

This article did not receive any specific grant from funding agencies in the public, commercial or not‐for‐profit sectors.

## ETHICAL STANDARDS

Not applicable.

## CONSENT TO PUBLISH

All authors agreed with the content and that all gave explicit consent to submit and publish the manuscript.

## Data Availability

Data sharing is not applicable to this article as no datasets were generated or analysed during the current study.
